# Time distortions in Alzheimer’s disease: a systematic review and theoretical integration

**DOI:** 10.1038/npjamd.2016.16

**Published:** 2016-09-08

**Authors:** Mohamad El Haj, Dimitrios Kapogiannis

**Affiliations:** 1 University Lille, CNRS, CHU Lille, UMR 9193-SCALab-Sciences Cognitives et Sciences Affectives, Lille, France; 2 Laboratory of Neurosciences, National Institute on Aging, Baltimore, MD, USA

## Abstract

Time perception is an essential function of the human brain, which is compromised in Alzheimer’s disease (AD). Here, we review empirical findings on time distortions in AD and provide a theoretical framework that integrates time and memory distortions in AD and explains their bidirectional modulation. The review was based on a literature survey performed on the PubMed and PsycInfo databases. According to our theoretical framework, time distortions may induce decline in the ability to mentally project oneself in time (i.e., mental time travel), and consequently may contribute to an episodic memory compromise in AD. Conversely, episodic memory compromise in AD may result in a loss of the ability to retrieve information about time and/or the ability to project oneself in subjective time. The relationship between time distortions and memory decline in AD can be jointly attributed to hippocampus involvement, as this brain area supports both time perception and memory and is preferentially targeted by the neuropathological processes of AD. Clinical implications of time distortions are discussed and directions for future research are suggested.

## Introduction

Time perception is a complex, yet essential, function of the human brain inter-twined with multiple cognitive processes. This ability is also fundamental for successful behavior in everyday life situations. Across different timescales, activities as diverse as scheduling a trajectory, preparing for a traffic light to change to red, and planning for an event, require execution of a sequence of actions towards a goal and are closely linked to time perception. On a psychological level, mental time travel, or the ability to relive the past and imagine the future, is thought to be at the heart of consciousness, and this extraordinary cognitive ability is possible thanks to time perception.^1^ Interestingly, this core ability is compromised in Alzheimer’s disease (AD).

AD is a progressive age-related neurodegenerative disease associated with distinct pathological changes (extracellular accumulation of amyloid-beta-containing plaques and intracellular development of tau-containing neurofibrillary tangles) in medial temporal and cortical regions.^2^ Clinically, AD is classically characterized by insidious and progressive episodic memory impairment,^2^ but disorientation in time is also a common early symptom in AD.^3^ Not surprisingly, cognitive assessment of AD patients tends to begin with evaluating their orientation in time; for instance, the Mini Mental State Examination,^4^ a widely used screening test for delirium and dementia, opens with five questions probing time perception. The clinical interest in time distortions in AD has been reflected by a body of empirical research showing timing distortions in perception of shorter and longer intervals, as well as in prospective timing (i.e., condition requiring conscious attention to the passage of time) and retrospective timing (i.e., condition involving incidental temporal processing). This body of research will be the main focus of this review.

**Figure 1:**
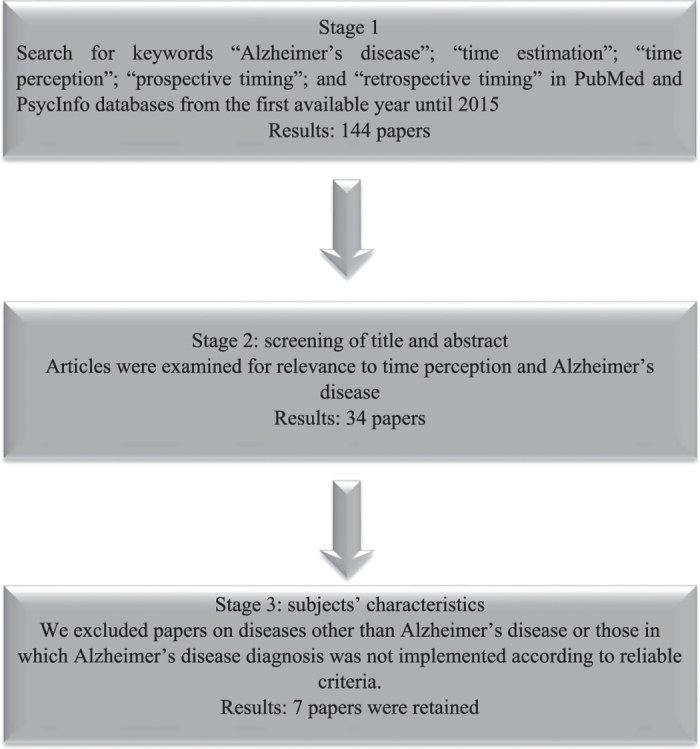
Overview of the selection process.

The following literature review was based on a literature survey that was performed by combining the keywords ‘Alzheimer’s disease’; ‘time estimation’; ‘time perception’; ‘prospective timing’; and ‘retrospective timing’. The search was performed on the PubMed and PsycInfo databases from the first available year until 2015; and was limited to studies published in peer-reviewed journals. Exploration of the reference lists of these papers was carried out to identify additional papers. We were careful to select papers in which AD diagnosis was implemented according to the reliable criteria, such as those of the NINCDS-ADRDA (National Institute of Neurological and Communicative Disorders and Stroke and the Alzheimer’s disease and Related Disorders Association) for probable AD.^2,5^ Hence, studies defining dementia according to general cognitive functioning rather than to reliable AD criteria were excluded. Figure 1 provides a flow diagram of the selection criteria and Table 1 summarizes the main results of the seven selected papers.

## Time distortion in AD: empirical research findings

In a pioneer study on time perception in AD, Nichelli *et al.*^6^ asked participants with AD to read either 5, 10, 20, or 40 digits appearing one at a time. At the end of each sequence, participants had to judge the elapsed time. Results showed inaccurate time estimations in AD participants, especially for long intervals. Inaccurate time estimation was also observed in another study in which participants with AD had to produce three empty intervals (5, 10, and 25 s); time production occurred by pressing a space bar on a keyboard at the beginning and at the end of each estimated interval.^7^ Similar findings were observed in a study by Rueda and Schmitter-Edgecombe^8^ who asked participants with AD to provide a verbal estimation of four time intervals: 10, 25, 45, or 60 s. During each interval, participants had to read aloud series of numbers that appeared on a computer screen. At the end of each interval, they had to answer the question ‘How long did the trial last?’ by providing a verbal estimation in seconds. Relatively to controls, time estimation of AD participants deviated significantly more from true clock time. Time distortions were also observed in a study by Caselli *et al.*^9^ who used time bisection tasks, during which participants with AD had to decide whether different time durations (ranging from 100 to 3,000 ms) were shorter or longer than a reference interval. AD participants showed specific difficulties in bisecting short intervals (100–600 ms).

Time distortions in AD may be associated with attentional compromise. This is suggested by Papagno *et al.*^10^ who asked participant with AD to provide verbal estimation of intervals during which they had to (1) utter irrelevant syllables, (2) press a key each time a ball entered a target square on a computer screen, or (3) perform a dual task. Each of the three tasks was performed during 15 and 50 s. Findings showed distortions in estimation of both intervals, especially during the dual task, which posed the greatest demand on attentional resources. The relationship between time distortions and attentional compromise can also be illustrated by a study in which participants with AD had to perform both a high and a low attentional task.^11^ On the high attentional task participants had to perform the interference condition of the Stroop test for 15 s, whereas in the low attentional task they had to fixate on a cross for the same length of time. At the end of each task, participants had to reproduce the duration of the previously viewed stimulus. AD participants under-reproduced the interval of the high attentional more than that of low attentional task; in other words, duration judgment decreased as task complexity increased. Thus, time perception in AD can vary depending on the attentional load of an ongoing task.

## Relationship between time deviations and memory compromise in AD

In the above mentioned literature, participants were either aware about time processing (i.e., prospective timing) or unaware about time processing (i.e., retrospective timing). A study by El Haj *et al.*^12^ assessed both prospective and retrospective timing by asking AD participants to read a series of numbers during four time intervals (30, 60, 90, or 120 s); before reading, participants were instructed that they had to provide, at the end of each trial, a verbal estimation of the elapsed time. In four additional tasks, participants performed four activities involving retrospective timing: deciding whether words were abstract or concrete (30 s), filling connected squares (60 s), deciding whether words were animal or object names (90 s), or reading a text (120 s). During these four tasks, participants were not informed about time judgment until they were asked to provide a verbal estimation of the elapsed time intervals. Besides time evaluation, the same study assessed mental time travel, i.e., the ability that allows humans to mentally project themselves backward in time, an ability closely associated with episodic memory.^1^ Mental time travel was assessed with the Remember/Know paradigm,^13^ during which participants had to provide a ‘Remember’ response if they could consciously recall details about a word presentation or a ‘Know’ response if they did not recall specific details about its presentation. ‘Remember’ responses reflect autonoetic consciousness, i.e., the ability to mentally project oneself back in subjective time to relive elements, whereas ‘Know’ responses reflect noetic consciousness, i.e., an abstract awareness of the past that does not include any recollection of specific experiences. The study of El Haj *et al.*^12^ showed decrease in the ‘Remember’ responses, suggesting compromised ability to mentally relive past events in AD, a decrease that was significantly correlated with distortions in prospective and retrospective timing.

According to Tulving,^1^ the most distinguishing characteristic of human memory is mental time travel, or the state of autonoetic consciousness permitting the episodic reliving of past experiences. By inducing the feeling of subjective time, mental time travel is likely to depend on time perception, which may explain why, in the work of El Haj *et al.,*^12^ the ‘Remember’ responses, reflecting autonoetic reliving, were significantly correlated with time perception. It is worth noting that, in the same study, no significant correlations were found between time perception and ‘Know’ responses, reflecting a dissociation between time perception and noetic consciousness.

In our view, time distortions may impair mental time travel, and consequently may contribute to an episodic memory impairment in AD. Conversely, episodic memory impairment in AD may result in a loss of the ability to retrieve information about time and/or the ability to project oneself in subjective time. For instance, due to anterograde amnesia, AD patients suffer from the inability to consciously acquire new memories, and as a consequence, are often ‘stuck in time’. The latter situation can also be experienced due to retrograde amnesia, which is observed with disease progression, where the inability to consciously retrieve stored memories may also result in a subjective experience of being ‘stuck in time’. Along these lines, there is now evidence that the time to reach a decision^14^ and the perception of time itself^15^ are influenced by the reliability (or uncertainty) of prior information that forms a prior probability signal. We speculate that, in AD, this reliability is degraded due to memory impairment potentially resulting in time distortions and longer decision times, collectively aggravating patients’ feeling of being ‘stuck in time’ and degrading their confidence in their decisions or actions.^16^ Furthermore, we speculate that the relationship between episodic memory and time perception impairment can be modulated in a bidirectional manner.

How memory can be activated by time processing can be illustrated with the attentional gate model,^17^ according to which time perception requires passing through an attentional gate-controlling pulses that are emitted by a pacemaker. When attentional processes are solicited for timing, the gate allows more pulses to head to an accumulator that counts the number of pulses, and this number represents the duration of an interval. When a target interval must be reproduced, the numbers of ongoing pulses that are counted into the accumulator are compared with previous pulses counts stored in working memory and long-term memory. Hence, memory is constantly invoked when reproducing target intervals. The attentional gate model was tested in normal aging and studies have demonstrated timing distortions in normal aging, especially for timing tasks that place high demand on working memory.^17,18^ The working memory involvement in time deviations in normal aging seems to be limited to decisions involving very short time intervals up to a few minutes in length.^19^ Deviations on longer time intervals appear to be governed by the mental representations of time. Studies highlight a particular mental representation of time in aging, i.e., time seems to pass more quickly as we age.^20^ For more than a century, philosophers and psychologists interested in humans’ consciousness have reported, and attempted to explain, why time appears to pass rapidly with aging.^21^ However, the impression of the speed of time with aging has been widely attributed to memory compromise.^20^ When older adults think about an interval of time (e.g., events that have happened since this time last year), they use to encounter difficulties to retrieved most of these events, which leads to the impression that the interval is relatively briefer than they may expect and consequently that time is passing quickly. This account is supported by empirical research showing that durations seem shorter if fewer events are recalled,^22^ an account of interest as it attributes time distortions to memory compromise in aging.

## Neuroanatomical correlates of the relationship between time distortion and memory impairment in AD

How the brain internally represents time is still debated although it is unlikely that it relies on a single amodal timekeeping region or circuit.^23,24^ Recent theories implicate spontaneous neuronal oscillators within multiple brain circuits that are calibrated by sensory information and feedback processes specific to each oscillator.^25^ Nevertheless, certain brain regions appear particularly important for time perception and processing.^23^ Neuropsychological research shows timing distortions in patients with frontal lesions,^6,26,27^ and neuroimaging studies show activation of the prefrontal cortex (PFC), particularly the right PFC, during processing of time (for a review and a meta-analysis, see refs 23 and 28). Specifically, the dorsolateral PFC is involved in the processing of brief intervals (<1 s),^29,30^ whereas the right lateral PFC and fronto-striatal circuits seem to be particularly involved in temporal foresight, similar to their involvement in the processing of long intervals.^31,32^ This timing function may be related to the critical role of (rostrolateral) PFC in sequencing tasks towards achieving a goal.^33^ Besides its involvement in time perception and action sequencing, the PFC is involved in mental time travel. Neuropsychological research suggests that individuals with damage to the prefrontal cortex have difficulties to project themselves in past or future.^13^ Also, the medial PFC appears to mediate the flexible use of memory information provided by the medial temporal lobe system, during mental time travel and remembering.^34^

**Figure 2:**
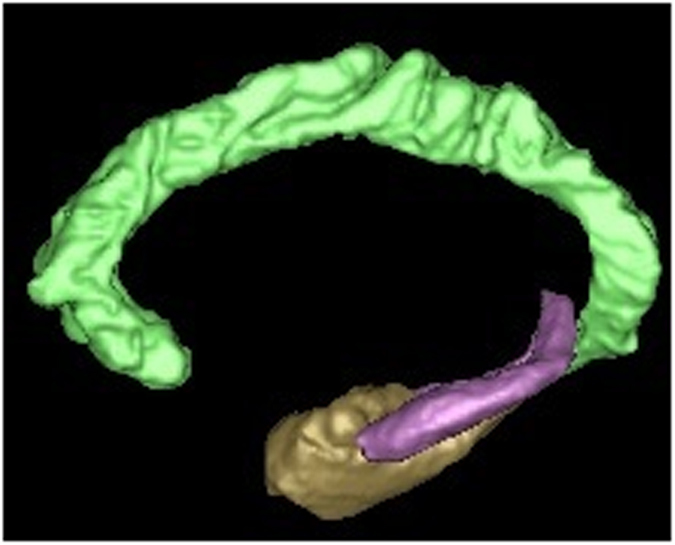
The hippocampus (here in purple) support both time perception and episodic memory. This brain area is preferentially targeted by the neuropathological processes in AD, which may support the relationship between time distortions and episodic memory decline in the disease. AD, Alzheimer’s disease.

Besides the PFC involvement, both time processing and memory depend on hippocampus functions (Figure 2). In the classic case of H.M., a patient who underwent bilateral medial temporal lobe resection, we observe not only an episodic memory compromise, but also underestimation for durations of more than 20 s.^35^ In a similar vein, a study has demonstrated distortions in prospective and retrospective timing in amnesic patients with left medial temporal lobe lesions.^36^ More precisely, timing difficulties and memory compromise can be related with compromise in the hippocampus. Studies highlight the involvement of the hippocampus not only in duration discrimination and representation of temporal sequences, but also in memory for elapsed time (for a review, see refs 37,38). Research studies have identified specialized ‘time cells’ in the hippocampus of rodents that support time stamping and binding of sequential events in memory.^39,40^ According to the latter research, the hippocampus is essential in retrieving the flow of events in distinct experiences, and in doing so, bridges the temporal gaps that occur in retrospective timing; more specifically, the hippocampus makes use of ‘time cells’ to recreate time frames for memory of elapsed time, contributing to subjective timing. Interestingly, the hippocampus, is preferentially targeted by the neuropathological processes of AD^41^ and its atrophy (especially to the right) underlies the episodic memory deficits observed in the disease,^42^ which may explain the relationship between time distortions and memory compromise in the disease. To the best of our knowledge, no published studies have investigated the neuroanatomical correlates of time distortions in AD, and more specifically, the hypothesized relationship between time distortions in AD and compromise of the hippocampus, or the prefrontal cortex. Therefore, the validity of this hypothesis remains to be determined by future research.

Unlike the substantial research on compromise of several cognitive functions in AD (e.g., memory decline, executive dysfunction), little research has been performed on time distortions in the disease. This is despite the clinical observation that disorientation in time is observed early in the disease and the fact it constitutes a cardinal feature of cognitive examination in AD (e.g., as in the Mini Mental State Examination of dementia). Although empirical research on time perception in AD confirms time distortions, the diversity of methods that have been so far employed makes it difficult to develop a comprehensive understanding of time distortions in the disease. Most of the reviewed studies have used verbal time estimation tasks,^6,8,11,12^ whereas few studies have used time reproduction^7^ or time bisection tasks.^9^ To better understand variations of time perception in AD, there is need for comprehensive studies to assess a wide variety of timing tasks in parallel. Future research should also assess time perception for long intervals (>60 s) as most studies so far have assessed intervals below this threshold. More empirical evidence is needed to delineate the relationship between time distortions and memory compromise in AD. For instance, it would be of interest to investigate relationships between time distortions and episodic autobiographical memory (i.e., memory for specific personal experiences), as the latter ability is heavily dependent on mental time travel^1^ and has been found to be compromised early in AD (for a review, see ref. 43). It would be also of interest to investigate whether time distortions are directly related with the ability to project oneself into the future, as the latter ability has also been found to be compromised in AD.^44–46^ In addition, it would be important to examine the relationship between time distortions, time to reach a decision, and degree of certainty, as all these processes rely on antegrade memory.^14,16^ Because we tend to consider the relationship between time distortions and memory compromise in a bidirectional fashion, it would be of interest to investigate how retrograde and anterograde amnesia may result in loss of temporal information in AD, and vice versa.

## Conclusion

Time is physically irreversible. This law can however be violated thanks to mental time travel. Traveling back into the past, a hallmark of human existence is compromised in AD, and this compromise can be associated with time distortions. Hippocampal (and perhaps PFC) damage may underline the compromise of both timing and memory functions in the disease.

## Figures and Tables

**Table 1:** Main findings of the seven reviewed studies

*Authors*	*Number of AD subjects*	*Time intervals*	*Main findings*
Nichelli *et al.*^6^	15	5, 40 s	Time distortions
Carrasco *et al.*^7^	8	5, 10, 25 s	Time distortions
Rueda and Schmitter-Edgecombe^8^	17	10, 25, 45, 60 s	Time distortions
Caselli *et al.*^9^	12	100–3,000 ms	Time distortions for short intervals (100–600 ms)
Papagno *et al.*^10^	21	15, 50 s	Time distortions
El Haj *et al.*^11^	17	15 s	Time distortions
El Haj *et al.*^12^	16	30, 60, 90, 120 s	Time distortions

Abbreviation: AD, Alzheimer’s disease.
